# Engineering nanoparticles to reprogram radiotherapy and immunotherapy: recent advances and future challenges

**DOI:** 10.1186/s12951-020-00629-y

**Published:** 2020-05-14

**Authors:** Jing Jin, Qijie Zhao

**Affiliations:** 1 Department of Oncology, The Affiliated Hospital of Southwest Medical University, Luzhou, 646000 Sichuan People’s Republic of China; 2 Laboratory of Molecular Pharmacology, Southwest Medical University, Luzhou, 646000 Sichuan People’s Republic of China; 3 Department of Pathophysiology, College of Basic Medical Science, Southwest Medical University, Luzhou, 646000 Sichuan People’s Republic of China; 4 South Sichuan Institute of Translational Medicine, Luzhou, 646000 Sichuan People’s Republic of China

**Keywords:** Nanoparticles, Radiotherapy, Immunotherapy, Immune checkpoint blockade therapy

## Abstract

Nanoparticles (NPs) have been increasingly studied for radiosensitization. The principle of NPs radio-enhancement is to use high-atomic number NPs (e.g. gold, hafnium, bismuth and gadolinium) or deliver radiosensitizing substances, such as cisplatin and selenium. Nowadays, cancer immunotherapy is emerged as a promising treatment and immune checkpoint regulation has a potential property to improve clinical outcomes in cancer immunotherapy. Furthermore, NPs have been served as an ideal platform for immunomodulator system delivery. Owing to enhanced permeability and retention (EPR) effect, modified-NPs increase the targeting and retention of antibodies in target cells. The purpose of this review is to highlight the latest progress of nanotechnology in radiotherapy (RT) and immunotherapy, as well as combining these three strategies in cancer treatment. Overall, nanomedicine as an effective strategy for RT can significantly enhance the outcome of immunotherapy response and might be beneficial for clinical transformation.

## Introduction

Nanotechnology has occupied worldwide attention in medical, chemistry, biology, and materials fields. In the oncology landscape, nanoparticles (NPs) were implicated in three main applications: drug vectorization, radiation-sensitization and medical imaging [1, 2]. The most popular and exceedingly used NP platforms are micelles, liposomes, polymeric NPs, and inorganic NPs [3–6]. Accordingly, nanomaterials have the properties to transport chemotherapeutic agents, radiosensitizers, oxygen storage agents and phototherapy agents, etc. Modified-NPs can successfully transport drugs across physiological barriers due to their high surface area, facile tunability and stability. Through enhanced permeability and retention (EPR) effect, NPs increases the accumulation of drugs in the tumor foci, including the classic radiosensitizers [7].

Radiotherapy (RT) is a mainstay strategy used to most tumor eradication or control. However, there is still a large challenge to enhance the therapeutic effects and reduce side effects [8]. In last decades, RT emerged as one of the most primary cancer treatment strategies, more than 50% of cancer patients have been participated in this treatment [9]. In the context of RT, the ultimate therapeutic benefit is to impede the tumor progression, while decreasing the additional risk of healthy tissue [9]. Moreover, NPs distribution and accumulation were up-regulated by the interaction between RT and tumor microenvironment (TME), which showed the exciting opportunity to enhance therapeutic benefit [10]. More recently, intensity modulated RT (IMRT), image guided RT (IGRT) and stereotactic ablative RT (SABR) have been considered as modern RT technologies, which are guideline-recommended accurate treatments to patients with mature and acceptable outcome [11, 12]. Besides, with a century of research on RT biological basis, 5 crucial factors were involved in determining the net effect of RT on tumors, including (1) cellular damage repairing; (2) repopulation ability of cells; (3) cell cycle redistribution; (4) cell reoxygenation; (5) radiosensitivity [13]. Modern therapy schemes are based on orchestrating these factors to boost tumor eradication, while reducing normal regions side effects. However, the cooperation radiobiological mechanisms were yet clear. NPs showed the positive ability to modulate these factors in tumor suppression treatment [14–16]. Furthermore, with appropriate radiosensitivity, NPs can control cells repopulation by ameliorating the immune responses in tumor milieu [17–19]. Owing to the development of nanotechnology, nanomaterials with heavy-metal showed a promising radiosensitization to enhance the favorable RT outcomes, such as gold and silver NPs, which can efficiently absorb, scatter, and emit radiation energy and were easily eliminated by metabolism [20, 21]. In addition, mesoporous silica, liposomes, bovine serum albumin (BSA) protein and polymeric were also used to deliver radiosensitizers to enhance RT effect [22–25].

Meanwhile, the delivery of certain chemical radiosensitizers by nanomaterials can improve their pharmacokinetic and pharmacodynamics, thereby promoting them to reach the tumor foci and enhance their anti-tumor responses [8]. Although the flourishing development of the NPs and RT, clinical translation remains a challenge, such as influence of nanoformulation properties, radiation sources selection, and complex tumor foci microenvironment [8]. Nevertheless, the strategy of combining RT and nanotechnology for cancer treatment still has a considerable promise in the future. Therefore, combining RT and nanotechnology has broad prospects in cancer treatment.

After RT, inevitable recurrence is still noted in 10–38% of patients and exhibits a higher risk of metastasis, which contributes to worse clinical outcome [26]. Strategies to prevent tumor recurrence is urgently needed. Recently, the underlying mechanisms behind post-RT recurrence were recognized [27], immune cells [T cells, Regulatory T cells (Tregs) and macrophages] and mesenchymal stem cells (MSCs) had evoked a great interest in TME [28–30]. By overcoming these shortcomings, the prime RT function in immune system to against cancer cells may harness the beneficial of local and abscopal effects. Moreover, pre-clinical researches in some tumors have demonstrated that localized RT combined with immunomodulation potentially unlocked the anti-metastatic and anti-relapse ability [3, 31–33]. It is imperative to utilize some optimized methods for patient with RT. Intriguingly, NP-based immunotherapy not only eradicates primary tumors and metastatic tumors, but also prevents relapse by immune memory reshaping [3].

Cancer immunotherapy comprised immunostimulatory monoclonal antibodies (mAbs), activatory cytokines, adoptive T cell therapy, cancer vaccines and microbiological adjuvants [34]. Synergistic combination of mAbs and/or immune checkpoint inhibitors provide multiple opportunities to modulate the intercellular communication against cancer, while the intensity of immune attacking response and eradication efficiency were two major synergy-indicators [34–36]. However, it still suffers from some limitations, such as dose-limiting systemic autoimmune side effects, limitative anti-tumor efficacy and benefits confined to certain subsets [37]. Herein, NPs can be an ideal carrier for eliciting and enhancing anti-tumor immunotherapy. Recent studies have indicated that combining immunotherapy with NP delivery system can boost antibodies accumulation and retention in the target cells [38, 39]. Accordingly, NP-encapsulated immune checkpoint inhibitors can improve immunotherapy response as well as reducing off-target effects. The high versatility of NP delivery systems could encapsulate different kinds of drugs, which can cooperate with immune checkpoint inhibitors to achieve better therapeutic benefits than immune checkpoint antibody alone [40]. Moreover, NPs designed for the modulation of macrophage polarization and reprogramming also play an important role in tumor immunotherapy [41]. Nowadays, the use of nanotechnology in combination with RT and immunotherapy evokes a novel avenue for overcoming current limitations as well as boosting cancer therapy effect [9]. In this review, we highlight the latest progress of nanotechnology between RT and immunotherapy. As well, we also provide a brief overview of the recent developments in applying these three strategies together to stimulate systemic anti-tumor immunity and obtain significant anti-tumor effects.

## Advancing radiotherapy through nanotechnology

**Fig. 1:**
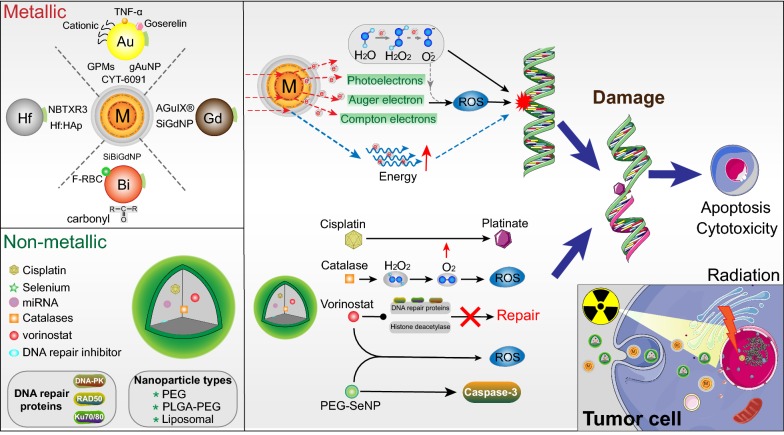
Schematic of nanoparticle functional mechanisms in radiotherapy. Combing ionizing radiation (IR) with nanoparticles (NPs) can boost radiosensitization, cell apoptosis and cytotoxicity. Upper: Metallic NPs (Au, Hf, Gd and Bi) deposit the IR dose through interactions, such as electron secretion (Compton, Auger and photoelectric), ROS generation and energy amplification. Down: Non-metallic NPs-encapsulated combined with radiotherapy further induced DNA damage and prevented rapid DNA repair, which will cause more cell apoptosis

Nanotechnology has enormous potential in cancer RT, which possesses inherent ability to selectively bind to cells. With EPR effect, the prior accumulation of NPs in the tumor sites could lead to some advantages: (1) enhancement for image-guided RT; (2) tumor-specific delivery of radiosensitizing drugs; (3) high atomic number (Z) particles can guarantee local dose of radiation [42]. High-Z nanomaterials can assimilate, scatter, and eradiate radiation energy, owing to the launch of low energy photoelectrons and Auger electron reciprocity [43]. Accordingly, released secondary electrons attack tumor cells and provide better treatment than radiation alone (Fig. 1). The released photoelectrons and Auger electrons can penetrate cells and hydrolyze water molecules to generate free radicals that interact with DNA, ultimately leading to DNA damage and cell death [44]. Literatures suggested that high-Z metallic NPs with the potential to cause radiosensitization, such as gold (Au), hafnium (Hf) and bismuth (Bi), gadolinium (Gd) (Table 1) [45]. Therefore, NP-enhanced radiosensitization was emerged as a classically adjunctive treatment strategy in cancers.

**Table 1:** Summary of studies on high-Z metallic nanoparticles radiosensitization

Element	Size/Zeta potential	Surface	Type	Cell line/model	Source energy	References
Au	13.5 ± 1.3 nm	Cationic polyelectrolytes	In vitro	Glioblastoma cells	40 kV, 80 mGy/min	[48]
	− 2.2 ± 0.5 mV	Polyethylene glycol-goserelin		PC3 cells	6MV	[50]
					X-ray, 5Gy	
	27 nm	Tumor necrosis factor-α		4T1/SCCVII cells	X-ray 12/20Gy	[51]
	75 nm	Polymeric micelles		HT1080 cells	X-ray, 4/6Gy	[52]
					150kVp, 6Gy	
Hf	25.2 nm	Hydroxyapatite	In vitro	A549 cells	662 keV, 5Gy	[61]
					662 keV, 5Gy	
Bi	56 nm	Folate plus red blood cell membrane	In vitro	4T1/HDF cells	115 kVp, 9Gy	[68]
	− 16.5 mV			4T1 cells	115 kVp, 9Gy	
Gd	3.5 ± 1 nm	Silica	In vitro	Capan-1 cells	220 kVp	[44]
	9 ± 5.5 mV				220kVp, 10Gy	

### Application of metal nanoparticles in radiotherapy

#### Au

Gold NPs (AuNPs) have the high X-ray absorption coefficient and easy to synthesize, the format of colloidal or clustered particles with Au core and surface coating can be precisely controlled by its special physicochemical properties [42]. As well, AuNPs showed the ability of specific radiosensitizers, ultimately eliciting and enhancing the tumors RT in pre-clinical research [46]. After the ionizing radiation (IR) is applied to biological system, the radiosensitization of AuNPs plays an important role in three phases, namely, physics, chemistry and biology, which determine the outcomes following IR [47]. Recently, Au-based multifunctional nanoplatforms invoked extensive interests in the biomedical field. As Zhang et al. [48] reported, cationic polyelectrolytes modified AuNPs promoted more DNA damage and cell death under the X-ray irradiation, compared to the negatively charged. Moreover, by increasing the accumulation of NPs in cancer cells with appropriate ligands, goserelin-conjugated AuNPs (gAuNPs) can enhance the local dose at megavoltage radiation energy range [49, 50]. Compared with RT alone, gAuNPs combined with RT showed significant radiosensitization and impeded tumor regrowth (17 ± 1 days) in heterotopic mouse, which mainly promoted short-range secondary electrons releasing, oxidative stress and cellular damage in prostate cancer [50]. In a Phase I clinical trial, gold-NP conjugated tumor necrosis factor-α (TNF-α), namely CYT-6091, significantly inhibited tumor growth and reduced interstitial fluid pressure when combined with radiation (12/20 Gy) in breast cancer and neck cancer models [51]. In addition, Zaki et al. [52] indicated that gold-loaded polymeric micelles (GPMs) modified NPs showed ability to increase circulation times, amplifying radiosensitization and tumor foci accumulation, which also provided a better CT image contrast for treatment monitoring. With these advantages, GPM-enhanced RT prolonged median survival time by 1.7-fold in mice models than radiation alone [52]. Thus, the AuNPs with different modifications can efficiently cooperated with cancer RT. Meanwhile, it also acts as a potential clinical contrast agent and radiosensitizer in amplifying the therapy effect.

#### Hf

Another man-made radioenhancer, Hf compound, is a high-Z nanomaterial with high-level electron density and deposites radiation energy in the tumor [53]. These NPs can develop many properties for optimal tumor cells uptaking, such as the shape, size and surface activities. In line with this, Hf with an atomic number of 72 can be made into hafnium oxide NPs (NBTXR3), which will produce more electrons at the same dose of radiation, thereby increasing DNA damage and subsequent cell death [53, 54]. Furthermore, in soft tissue sarcoma (STS), a phase I study of the first human trial demonstrated that NBTXR3 plus external beam RT (EBRT) can achieve a 40% median tumor shrinkage rate and 26% median percentage of residual malignant cells rate [55].

Recently, there has a multicenter, randomized, phase I/II clinical trial in STS, which illustrated that NBTXR3 enhanced radiosensitization in RT (NCT02379845). 176 eligible patients with locally advanced STS were randomly divided into two groups: EBRT (50 Gy in 25 fractions) alone and NBTXR3-mediated EBRT (a single intratumoural administration). The pathologic complete response rates of the NBTXR3 group and EBRT alone group were 16% and 8%, respectively. While, the trail data also showed some emergent side-effects, and nearly 39% patients in the NBTXR3 and 30% in RT group had serious adverse events, such as postoperative wound complication, injection site pain, hypotension and radiation skin injury [56]. In consistent with other NBTXR3 clinical evaluated in cancer, although it has some complications, it can effectively improve RT and patients could benefit from the treatment (NCT01946867&NCT02901483/head and neck; NCT02721056/liver; NCT02805894/prostate; NCT02465593/rectal) [56]. In the course of treatment, when metallic NPs were exposed to IR as sensitizers, there will have more compton electrons, secondary electrons, and photoelectrons emitting [57]. Intriguingly, these enhanced electrons can interact with water molecules, and locally produce free radicals to trigger the quantity of reactive oxygen species (ROS), which predicted radical-induced DNA strand damage and highly cytotoxic in tumor cells [58, 59]. Once NBTXR3 has accumulated in tumor foci, it will generate large quantities of electrons with the ionizing radiation, by which, cancer cells eradication function and healthy tissues protection were enhanced [54, 60].

Moreover, Chen et al. [61] indicated that joint Hf ions into hydroxyapatite (Hf:HAp) showed a capability to yield large quantities of ROS in the cells when it was exposed to IR. The HAp was previously used to improve imaging contrast cell separation and drug delivery in cancer treatment, and also was considered to be an ideal host material for biological applications [62, 63]. Hf:HAp-triggered ROS was consistent with cytotoxicity and anti-tumor efficient, which opened a novel window for synthesized material in cancer RT [61]. Together, Hf as a special nanomaterial that can be used to cancer palliative treatment and potentially converted into clinical benefit.

#### Bi

Especially, as a most biocompatible radiosensitizer with biggest atomic number, Bi has ability to maximize radiation absorption efficiency and sensitivity, which has been used clinically for many years [64, 65]. The synthesis of Bi NPs has biodegradable properties and can be exhausted from the body in the form of soluble Bi ions [66]. Otherwise, non-degradable NPs may accumulate inside body and cause serious side effects. In order to overcome the bottleneck of monodispersity, coproduct and quality, various methods have been developed to synthesize Bi NPs, such as solvothermal method, photochemical method, and precursor method [67].

Recently, Bi NPs modified with folate plus red blood cell membrane (F-RBC) had invoked great interesting in breast cancer RT, especially the ability of triggering ROS production to tumor damage and fine clearance ratio [68]. After the sensitized radiation treatment in 4T1 tumor-bearing mice, F-RBC Bi NPs significantly promoted tumor regression and prolonged survival, meanwhile, it was completely excreted from body after 15 days [68]. Similarly, in breast cancer, cellulose nanofibers were used as templates to make ultra-small Bi NPs, which also boosted the secretion of ROS in the presence of X-ray radiation and showed high cytotoxicity to tumor [69]. Due to the rich surface carbonyl groups in nanofiber, it can effectively absorb Bi and prevent local oxidization to make sure the biocompatibility and inhabitation. In order to improve the anti-tumor effect and image contrast accuracy, the multi-metal overlapped NPs were proposed, such as ultrasmall silica-based bismuth gadolinium NPs (SiBiGdNP). As a novel trimodal theranostic NP, intravenous administration of SiBiGdNP can amplify the dose of radiation under clinical exposure conditions, which also significantly improved tumor DNA damage, tumor regression and survival, compared to RT alone [70]. On the contrary, no crucial increase of DNA double-strand breaks was observed in healthy tissues compared to control group. For the scheme of next-generation radiosensitizers, multi-metal NPs synthesis may pave a way for the precise control and monitoring of RT in tumors. In this approach, multi-metal NPs may generate variations in secondary Auger electron spectra and translate it into biological effectiveness, ultimately maximizing treatment effect and minimizing toxicity [71].

#### Gd

Gd-based NPs can enhance the radiation dose, as well as becoming magnetic resonance (MR) contrast agent [44]. Interestingly, silica-based gadolinium chelated NP (SiGdNP), a ultrasmall NP (~ 1–5 nm), can promote efficient radiation-induced DNA double-strand breaks with 6 MV [44]. Thus, the synergy between Gd NPs and RT not only depended on agents, but also was associated with inherent physicochemical properties. Following this, SiGdNP was highly accumulated in the tumor via the EPR effect, and MR imaging (MRI) and irradiation can be benefit [72]. Meanwhile, SiGdNP radiosensitization boosted the tumor regression and overall survival (OS) compared to RT alone in the human pancreatic xenograft model (SiGdNP +/IR + 2.69 ± 0.16 vs SiGdNP-/IR + 5.32 ± 0.19 cm^3^) [44]. Similarly, Gadolinium-based NPs (AGuIX^®^), combined with irradiation both in vivo and vitro, which induced tumor cells death and prevented tumor metastases, thereby increasing the life span to 25% compared with 8.3% of irradiated alone [73]. AGuIX^®^ is an effective T1-MRI contrast agent due to the presence of Gd, acting as the imaging agent and radiosensitizer, simultaneously [73]. Together, there are promising potential for high-Z NPs to attain clinical translation, so it is urgent to continue interdisciplinary research to translate such NPs radiosensitizers into clinical practice.

### Nanoparticle-encapsulated radiosensitizer

In addition to metallic nanomaterials, NPs also were used as delivery vehicle for tumor-specific radiosensitivity drugs, such as chemical radiosensitizers, siRNAs, microRNA (miRNA), oxygen carriers, catalases and so on (Fig. 1) [8, 74]. Strikingly, the strategy of nanocarrier delivering radiosensitizers [cisplatin, Selenium (Se), DNA repair inhibitor, catalases, and miRNA] has a promising prospect. The advance of multifarious nanocomplexes facilitated the development of radiosensitizers in tumor therapy. Moreover, previous studies have demonstrated that various NP forms were utilized to deliver radiosensitizers, such as liposomes, mesoporous silica NPs, bovine serum albumin protein NPs and polymeric NPs [22–25]. Recently, nanotechnology progresses have supplied new hope for next-generation cancer treatment. To overcome the antineoplastic obstacle in clinical translation, some NP-encapsulated chemotherapeutic drugs have been clinically recognized and effective compared to unencapsulated forms, such as doxorubicin (DOXIL, Caelyx, Myocet), paclitaxel (Abraxane), irinotecan (Onivyde) and vincristine (Marqibo) are clinically approved nanoformulations [75]. Additionally, RT can help drug-loaded NPs delivery via the effects on tumor-associated immune cells, vessel enlargement and interstitial fluid in the tumor [10, 75]. In particularly, tumors with large numbers of macrophages are usually difficult to treat, but combination of ionizing radiation and drug-loaded NPs might be a potential approach in such cases [76]. However, there are still some limitations yet to be solved, including neighboring tissue damage, radioresistant and hepatic clearance [75].

In recent, NPs containing cisplatin have provoked great interests in tumor therapy development. Previous clinical data indicated that cisplatin is one of the most powerful chemotherapy drugs for effective cancer chemo-radiotherapy [77]. By binding with cellular DNA, cisplatin concentrated radiation in the vicinity of targeted DNA and contributed to DNA damage [78]. Herein, NP coated with cisplatin prodrug will produce platinated in cell nuclei and make DNA more vulnerable. Concomitantly, radiosensitivity will be amplified [79]. Moreover, multi-drug NPs were also proposed to enhance the tumor cells apoptosis in RT. In addition to cisplatin-induced DNA lesion, co-delivered vorinostat will parallelly prevent DNA repairing, which inhibited the histone deacetylase, DNA repair proteins (Ku70/Ku80, DNA-PK, and RAD50) [80], and produced ROS to delay the repair of DNA double-strand [79]. Because ionizing radiation induced DNA damage can subsequently active the initiate DNA repair network [81]. This dual-targeting NP radiation therapy strategy to enhance tumor cells apoptosis by depositing irradiation energy and targeting DNA double-strand breaks showed the potential to improve clinical outcomes [79]. In line with this, Zhang et al. [82] indicated that co-delivery of cisplatin and wortmannin (DNA repair inhibitor) by Food and Drug Administration (FDA)-approved PLGA-PEG (polylactic acid-glycolic acid copolymer-polyethylene glycol) NP made a great success in suppressing the tumor growth by precisely coordinating with RT, in both platinum-sensitive ovarian cancer (PSOC) and platinum resistant ovarian cancer (PROC) murine models, compared to free drugs or single-drug loaded NPs. Additionally, PLGA-PEG NP not merely acted as vehicle to deliver Wtmn and cisplatin, but also showed the ability to overcome cisplatin-resistance, minimize side effects, promote cisplatin accumulation, improve Wtmn stability and boost therapeutic efficacy [82]. Meanwhile, sufficient molecular oxygen is essential for radiation-induced DNA breaks. [83]. Relieving tumor hypoxia plays a significant role in improving cancer RT. Recently, several studies identified that antioxidant enzyme (catalase) could decompose hydrogen peroxide (H_2_O_2_) into H_2_O and O_2_, which maybe an effective route for improving tumor reoxygenation and hypoxia-associated radiation resistance [84–86]. Furthermore, liposomal NP coated with cisplatin prodrugs and catalase enhanced RT effect and impeded tumor growth, which alleviated cellular hypoxia by triggering the decomposition of H_2_O_2_ [87]. Severe morphology changes and necrosis were obtained by H&E staining compared to the control group. Within this multifunctional nanocomposite features, several advantages were observed: (1), fully biocompatible; (2), high transport efficiency; (3), reversing hypoxia associated radiation resistance [87]. Strikingly, NPs co-delivered with cisplatin and these radiosensitizers are expected to be clinically translated in synergistic cancer RT.

Nowadays, selenium NPs (SeNPs) have attracted extensive attention as a potential anti-tumor agent and drugs carrier, which exhibited excellent antioxidant ability, favorable biocompatibility, lower toxicity and cancer prevention effects [88–90]. In this approach, SeNPs may overcome the sharp decline of plasma Se level in clinical RT [91]. As a trace element, Se has novel photoconductivity, piezoelectricity, nonlinear optical response and pyroelectricity [92]. The role of Se in chemotherapy has been extensively studied. Simultaneously, SeNPs can stimulate ROS production and possess broad spectrum of anti-cancer activity [93, 94]. Furthermore, SeNPs were good candidates to replace other types of selenium in pharmaceutical dosage utilizing, because nano-size elemental selenium (Se0) show a much lower toxicity than selenite (Se^+2^ or Se^+4^) ions [95]. As a new radiosensitizer, SeNPs combined with irradiation treatment elevated tumor cell killing effect and reduced normal tissue damage, which especially reinforced radiosensitivity, G2/M phase cell cycle arrest, and autophagy activation [96]. Irradiation treatment (6 Gy) combined with SeNPs (3 ug/ml) increased cell death up to 29.67% and apoptosis up to 14.94% (versus SeNPs or 6 Gy irradiation alone) [96]. Apart from irradiation-induced cell death, autophagy has also become a significant channel for tumor eradication by irradiation, indeed, it helped to aggravate tumor metabolic stress and contribute to cell death and proliferation inhibition [97]. Herein, SeNPs simultaneous targeting ROS, cell cycle apoptosis and autophagy appeared to further strengthen tumor RT efficiency [97]. The PEG modified SeNPs (PEG-SeNPs) made a great progress in amplifying the irradiation treatment, because its amorphous characteristics elicited significant radiosensitization [94]. Meanwhile, the compound in the outer layer of PEG can prolong blood circulation time after interventional therapy [98]. Co-treatment of cancer cells with PEG-SeNPs and irradiation significantly suppressed the cells growth by inducing cell apoptosis, which was verified by DNA fragmentation, ROS overproduction and caspase-3 activation [94]. Consistent with this, in 2018, the combination of PEG-SeNPs and irradiation treatment showed up-regulated caspase-3 activity and higher tumor cells apoptosis in lung cancer [99]. After irradiation treatment, PEG-SeNPs also significantly generated large amount of ROS in lung cancer cells and induced ROS-mediated apoptosis [99]. Together, novel strategy of combination of nano-delivered radiosensitizers and irradiation might be an effective chemo-radiotherapy.

## Nanotechnology to improve immune checkpoint blockade

Immune system interacts with tumor initiation, progression, invasion, and metastasis, and it makes cancer possess another dimension of complexity. As a hallmark of cancer, complicated crosstalk between cancer cells and the immune system is a double-edged sword: it enhances or suppresses tumor growth [100]. Recently, cancer immunotherapy emerged as a novel cancer treatment method, and significant advances have been made clinically. Based on the development of basic cancer immunology and translational immunotherapy, adoptive cell therapy (ACT) and immune checkpoint blockade therapy have a major impact on patients with advanced cancer [101, 102]. While, the approaches of genetic engineering, drug delivery and nanomedicine fields invoked a maximized potential of immunotherapy [103]. Targeting tumor-specific cells rather than the entire lymphocyte compartment non-specifically [104]. Thus, there are both opportunities and challenges for the delivery system to tumor immunotherapy, especially NPs biotechnologies. More importantly, immune checkpoint intervention plays an important role in maintaining immune homeostasis and preventing T-cell exhaustion, which can regulate the tumor immune microenvironment [105]. Immune checkpoint inhibitors have a potential property to improve clinical outcomes in cancer immunotherapy [106, 107], such as programmed cell death protein 1 (PD-1)/programmed cell death-ligand 1 (PD-L1) axis, cytotoxic T lymphocyte antigen-4 (CTLA-4), indoleamine 2,3-dioxygenase (IDO), cluster of differentiation 40 (CD40), as well as 4-1BB (CD137). For example, by recovering T-cell function and facilitating cytotoxic T lymphocytes (CTLs) responses, CTLA-4 immune checkpoint blockade therapy can enhance the host’s immune system [108]. However, low durable response rates and side effects of immunological checkpoint inhibitors are the reasons for the limited clinical application of immune checkpoint blockade therapy [40].

**Fig. 2:**
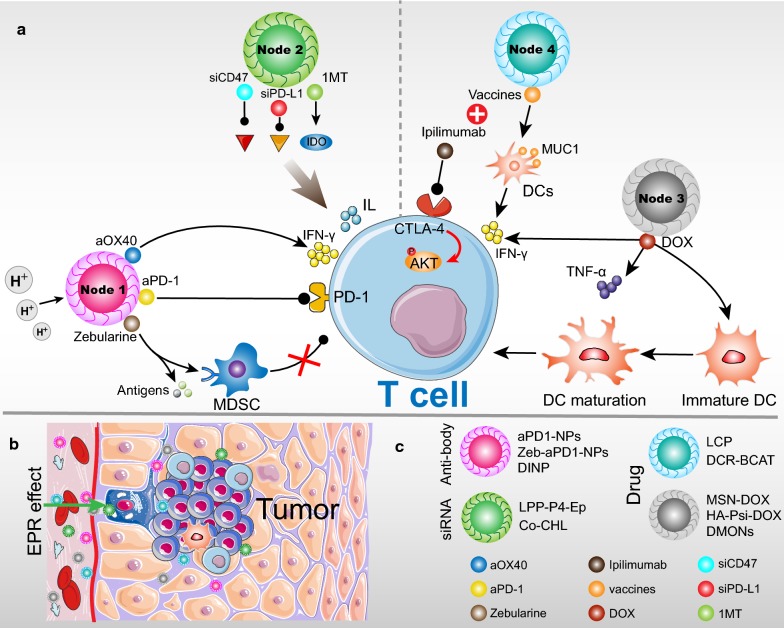
Targeting immunomodulators to immune cells in tumor microenvironment (TME). **a** Strategies for the application of NPs in immune checkpoint blockade therapy: NP-encapsulated immune checkpoint inhibitors and PD-L1 siRNA (Node 1 and 2), NP-encapsulated different drugs combined with immune checkpoint inhibitors (Node 3 and 4). The particles carried the target modulator, which further targeted the epitopes on immune cells, and direct or indirect antitumor action. **b** NPs extravasate into the tumor stroma through the fenestrations of the angiogenic vasculature, which can be enhanced by the EPR effect. **c** NPs have ability to carry one or more therapeutic agent to modulate T cells activation. Upper: Category of NP assembly; Lower: Different therapeutic payload

The multidisciplinary nanotechnology for the targeted delivery of immunoregulatory and imaging contrast agents provided an anti-tumor breakthrough [109, 110]. Strikingly, nano-delivery systems have made considerable progress in combination with immunotherapy agents [111], which increased the targeting and retention of antibodies in target cells with EPR effect [112]. Through the EPR effect, macromolecules and NPs are prevented from being removed from the tumor and passively target the tumor, which is regarded as effective agent design standard (Fig. 2b) [113]. NP-payload immune checkpoint inhibitor can improve antibody accumulation, upregulate the immunotherapeutic responses, and enhance the effective delivery, while decrease the off-target effects [40]. Simultaneously, targeting delivery of PD-L1 siRNA to the immunosuppressive pathway has recently been studied. Compared to antibodies, siRNA can enhance antitumor immune responses and reduce the side effects in some extent [114]. At last, NP-encapsulated different drugs combined with immune checkpoint inhibitors also can improve anti-tumor effect, compared with immune checkpoint antibodies alone [115]. These related NPs-triggered immunomodulation and related cytokines are presented in Table 2.

**Table 2:** Nanoparticle immunomodulation information and related cytokines

Nanoparticles	Immune checkpoint inhibitor	Anti-tumor immune activation	Cytokines secretion	References
Zeb-aPD1-NPs-Gel	Encapsulated aPD1	CD8^+^/CD4^+^ T cells	Unknown	[117]
DNIP	Co-encapsulated aPD1 and aOX40	CD8^+^ T cells	IFN-γ	[123]
Co-CHL	Encapsulated si-PDL1	CD8^+^ T cells	IFN-γ, IL-2	[130]
LPP-P4-Ep	Encapsulated si-PDL1	T cells	IFN-γ, IL-4, IL-6, IL-10	[137]
HA-Psi-DOX	Combine with aPD1	CD8^+^ /CD4^+^ T cells	IFN-γ, TNF-α	[143]
DMONs	Combine with aPDL1	CTLs	IFN-γ, TNF-α,IL-12, IL-1β	[149]
LCP NPs deliver MUC1 mRNA vaccine	Combine with CTLA-4	CD8^+^ T cells	IFN-γ	[154]

### Delivery of checkpoint inhibitors

Delivery of checkpoint inhibitors with NP can improve the durable response rate of T cell-based immunotherapy (Fig. 2a; Node 1) [40, 116]. In one study, anti-PD-1 (aPD1) loaded in the pH-sensitive CaCO3 NPs (aPD1-NPs) showed increased accumulation and locally sustained release in tumor foci, which will disintegrate and release aPD1 to react with H^+^ [117]. Simultaneously, co-encapsulated aPD1-NPs with Zebularine (Zeb) engineered in ROS-responsive hydrogels (Zeb-aPD1-NPs-Gel) effectively elicited the immunogenicity and reversed immunosuppressive in tumor cells. Zeb is a hypomethylating agent to inhibit proliferation and trigger apoptosis, which also plays a pivotal role in regulating tumor immune microenvironment [118, 119]. Interestingly, hypomethylating agent like azacytidine was previously reported to induce immunosuppressive ligands expression and immune checkpoint blocking therapy sensitivity, including PD-L1/PD-L2 axis and CTLA4 [120, 121]. In present study, the co-delivery of Zeb to aPD1-nanoparticle elicited a stronger anti-tumor effect, like stimulating PD-L1 and tumor-associated antigen expression, and reversed the tumor immunosuppressive by reducing myeloid-derived suppressor cells (MDSCs) [117]. Meanwhile, Zeb-aPD1-NPs-Gel group significantly inhibited tumor growth and obtained longer median survival time to 39.5 days compared to other groups (untreated group 16 days, Zeb-NPs 16 days, aPD1-NPs 18 days, and aPD1-NPs Zeb 23 days). Mechanically, the rate of CD8 T-cell infiltration in tumor group treated with Zeb-aPD1-NPs-Gel was 4.5% of total tumor cells by flow cytometry and immunouorescence analysis, which was 1.9-fold of aPD1-NPs Zeb group and more than 2.73-fold of Zeb-NPs-Gel group. Additionally, the number of activated CD8^+^ T cells (CD8^+^ CD44^+^ T cells) was significantly increased in the Zeb-aPD1-NPs-Gel treatment group, thereby enhancing the function of CD8^+^ T cells. Overall, this co-delivery system can availably enhance T cell-mediated anti-tumor immune responses [117].

In recent study, precise NPs combined two immunotherapeutic regimens to enhance the therapeutic efficacy: blocking T-cell suppression (aPD1) and inducing T-cell activation (aOX40). Meanwhile, a combination of aPD1 and aOX40 agents was proposed to boost T-cell activation [34, 122]. Mi et al. [123] demonstrated that PEG-PLGA NP coupled with aPD1 and aOX40, named dual-immunotherapy NP (DINP), had synergistic tumor immunotherapeutic effects to invoke T-cell activation at a rate higher than free antibodies. The DINP surface is negatively charged and has an average hydrodynamic diameter of 166.9 ± 6.5 nm. IFN-Enzyme-Linked ImmunoSpot can assess T cells activity [124], and DINP-treated group presented a higher overall activity of IFN-γ and more cells that produced IFN-γ. In line with this, the DINP treatment group showed a higher percentage of T-cells than free antibody treatment arms in tumor model (20.1% vs 4.9%). Importantly, DINP approach also showed an advantage to preserve durable antitumor immunological memory than conventional antibody therapy, and mice bearing bilateral flank B16-F10 melanoma treated with DINP group has achieved cure rate of 30% and 5/6 of the cured mice successfully resisted tumor recurrence (OS: DINP 30% vs antibody alone 10%). In addition, this therapeutic character was also available on the orthotopic 4T1 breast cancer model. Compared to mixture of free aPD1 and aOX40 in melanoma mice, the DINP treatment group represented larger amount (median: 19.0 vs 6.9) and more infiltration of CD8^+^ T-cell (85.2% vs 68.5%). Additionally, among the activated CD8^+^ T-cell, DINP induced effector memory T-cell frequency was two-fold higher than antibody regimen (median: 54.4 vs 23.0) [123]. Tumor-specific effector T cells had been regarded as an important biomarker for human melanoma survival and immunotherapy effect, recently [125]. Taken together, co-delivery of immune checkpoint inhibitors by DINP can stimulate more effective T-cells activation than antibody or therapeutic NPs alone, thereby forming a high central-memory ratio microenvironment to improve immunotherapy efficacy [123].

### Target delivery of PD-L1 siRNA

As an alternative strategy, siRNA nanotechnology was also used to stimulate higher immune response (Fig. 2a; Node 2). Delivering of ideal PD-L1 siRNA (siPD-L1) targeted the immune checkpoint significantly induced higher antigen-specific T-cell response and tumor regression [17]. Specifically, using siRNA to block PD-L1 and weaken PD-L1/PD-1 axis interaction can avoid antibodies off-target effects related normal tissues immune-related adverse events (irAE), such as skin, gastrointestinal, and respiratory systems [126, 127]. siPD-L1 can specifically block the synthesis of new PD-L1 to liberate T-cell co-stimulatory receptor, because extensive expression of PD-L1 had been observed in TME [127, 128]. More recently, Guorui et al. [129] proposed that siPD-L1 and 1-methyl-DL-tryptophan (1MT) (indoleamine 2,3-dioxygenase inhibitor) packaged in the nanodelivery system (Co-CHL) can efficiently accumulate and release in tumor foci, which contributed to cytotoxic T lymphocytes activation and tumor cells apoptosis [130]. Accordingly, both siPD-L1-nanoparticle and Co-CHL significantly inhibited PD-L1 expression in 4T1 tumor cells, while Co-CHL induced higher tumor cells apoptosis compared with siPD-L1-nanoparticle alone. It showed that combined siPD-L1 and 1MT in NP not merely target delivered the immune checkpoint blocking drugs, but also obtained synergistic enhancement of tumor cells apoptosis ratio. Through the T cell-based anti-tumor mechanisms investigation, compared to siPD-L1 or 1MT control group, Co-CHL treatment contributed to more numbers of intratumoral CD8^+^ T cells, and higher CD8^+^/CD4^+^ T-cell ratio, resulting in stronger tumor regression and higher therapeutic efficacy. Moreover, the Co-CHL group IFN-γ and IL-2 were significantly upregulated (reached 151.67 ± 5.50 and 57.33 ± 2.50 pg/mL, respectively) compared to the negative control and blank control, both of which were key positive markers of T-cell activation and proliferation [130–132]. Thus, based on enhanced function of the T cells and immune microenvironment, this novel strategy can conquer the immune escape mechanism of tumor cells.

PD-L1 is highly expressed on the surface of tumor cells [133], and CD47 (Integrin-Associated Protein) is expressed in tumor cell-surface to serve as an immune checkpoint [134], both of which can promote tumor immune evasion. Previously, anti-CD47 and anti-PD-L1 dual-blocking achieved an enhanced therapeutic efficacy in melanoma and colon carcinoma immune checkpoints inhibition treatment [135, 136]. The undesired side effects still remain a question. However, EpCAM-targeted cationic liposomes (LPP-P4-Ep) NP co-delivery siCD47 and siPD-L1 was designed to avoid this defect and enhance immune therapeutic efficacy [137]. The particle size of LPP-P4-Ep is about 175 nm, and zeta potential is 37.1 Mv. Through inhibiting CD47 and PD-L1 proteins expression, double RNA interference indicated a better survival rate and more effective immunotherapy. After LPP-P4-Ep NP treatment, PD-L1 and CD47 were effectively silenced. Moreover, blocking PD-L1/PD-1 and CD47/SIPR-α axes subsequently induced monocytes to secrete IL-6 and IFN-γ, thereby regulating immune response. In vivo, LPP-P4-Ep-treated mice have the higher percentage of T cells, natural killer(NK) cells and immune cytokines (IFN-γ, IL-4, IL-10 and IL-6) compared to the control group [137]. These cytokines are significant for immune response [138, 139]. LPP-P4-Ep group also reduced tumor volume by 87% in mice bearing 4T1 breast cancer tumor and decreased lung micro-metastasis in 4T1 metastasis model, compared to the untreated group. Furthermore, modified nanocarriers addressed side effects related to antibody therapy [137]. Consequently, NP-based siPD-L1 might be a novel strategy in immunotherapy, inducing anti-tumor immune responses and avoiding the side effects of antibodies.

### Nanomedicine combined with checkpoint inhibitors

Nano-platform delivery of different drugs combined with immune checkpoint inhibitors presented a promising cancer immunotherapy [140]. Despite the high efficiency of immune checkpoint blockade on tumor eradication, only minority of patients response ideally to this treatment [141]. Accumulating evidences revealed that checkpoint blockade treatment mainly benefited from patients whose tumor have pre-existed local CD8^+^ T-cell infiltration [140, 142, 143]. It limited the using of immune checkpoint inhibitors in clinical scope. According to recent literature, chemo-immunotherapy combination was proposed to ameliorate the checkpoint blockade therapies defect by using chemotherapy drugs to induce anti-tumor immune response [144].

Doxorubicin (DOX), a widely used systemic chemotherapy drug, which induced so-called “immunogenic cell death (ICD)” process, stimulating anti-tumor immune responses and increasing antigen-specific T cells activation, proliferation, and infiltration (Fig. 2a; Node 3) [145]. The Mesoporous silica NPs (MSN) encapsulated DOX (MSN-DOX) showed ability to promote dendritic cells (DCs) maturation and anti-tumor cytokines releasing [144]. In addition, MSN-DOX is suitable for imaging guided targeting therapy and obviously accumulated in the tumor foci. The stimulated anti-tumor immune response by MSN-DOX also ensured higher tumor-infiltrating CD8^+^ T-cell level. An ideal cell killing efficiency was presented by this reliable approach. However, DOX chemotherapy not only initiated antitumor immune response, but also stimulated the secretion of INF-γ [143, 146]. Upregulated INF-γ will improve PD-L1 expression level in tumor, which can interact with PD-1 and contribute to immunosuppression [147]. Herein, Gao’s group established a matrix metalloproteinase-2 (MMP-2) sensitive hyaluronic acid-PLGLAGG-doxorubicin prodrug (HA-Psi-DOX) combined with anti-PD-1 to simultaneously provoke antitumor immune response and neutralize immunosuppression function, which can accumulate at the tumor foci by EPR effect and has low systemic toxicity [143, 148]. This spherical NP is approximately 70 nm in transmission electron microscope (TEM) image. HA-Psi-DOX has significantly toxic to B16F10 cells with high expression of MMP-2 and leads to the “ICD” process, ultimately eliciting an anti-tumor immune response [143]. Meanwhile, IFN-γ content and PD-L1 expression induced by HA-Psi-DOX were 1.84-fold and 1.4-fold higher than free DOX, respectively. When combined with anti-PD-1, mice treated with HA-Psi-DOX increased survival rate and reduced the tumor volume and metastasis, which may be linked to the robust DOX-induced TILs recruitment in tumor beds [143]. In addition, another nano-packaged DOX is also designed for precisely target immuno-suppressive tumors. With the dendritic mesoporous organosilica NPs (DMONs), DOX and PD-L1 antibody synergistically enhanced chemo-immunotherapy compared to other control [149]. Importantly, increased level of tumor necrosis factor alpha (TNF-α) was also observed after DMONs treatment, which is an important marker for anti-tumor immunity [149]. Together, the utilization of suitable nanomedicines will pave a way in regulating superior anti-tumor immune microenvironment.

CTLA-4-targeting antibody is another checkpoint inhibitor. CTLA-4 is a CD28 high homology gene, it will recognize the B7 molecule of T-cell/APC interface [150, 151]. Based on the knowledge of CTLA-4, initiated by T cells, it was first thought to be a costimulatory molecule [152]. However, accumulation of CTLA-4 on T-cell/APC interface eventually contributes to costimulatory blocking and T-cell responses suppression [147]. Ipilimumab, an antibody against CTLA-4, had approved by FDA for the treatment of melanoma in 2011, resulting in stimulating effector T-cell and depleting Tregs in tumors [153]. By targeting T cells regulatory pathways, CTLA-4 monoclonal antibody has been successfully combined with mRNA vaccines in lipid/calcium/phosphate (LCP) NPs, it significantly improved anti-tumor immune response than vaccine alone (Fig. 2a; Node 4) [154]. Moreover, major histocompatibility complex (MHC)-I restricted CTLs plays an important role in eliminating tumor cells and prevents cancer recurrence [155]. Mannose-modified LCP-NPs promoted the delivery of mRNA vaccine encoding tumor antigen MUC1 to DCs, thereby inducing MHC-I restricted CTLs responses [154, 156]. The result indicated that target antigen MUC1 up-regulated the killing efficiency of antigen-specific CD8^+^ cells and induced the IFN-γ production. Not surprising, the MUC1 vaccine combined with anti-CTLA-4 monoclonal antibody can induced more tumor-infiltrating CD8^+^ T cells than single treatments [154]. CTLA-4-blocking leaded to AKT phosphorylation, ultimately promoting T-cell activation [157], which locally enhanced the therapeutic effect when used in combination with MUC1 vaccine NP. Similarly, Ganesh et al. [158] also developed an RNAi NP (DCR-BCAT) that targeted the gene encoding β-catenin, which can increased the infiltration of T-cell and enhanced the sensitivity of tumors to immune checkpoint inhibitors. Wnt1-driven tumors achieved complete tumor regression when DCR-BCAT treatment was combined with CTLA-4 or PD-1 antibodies [158]. Overall, NP-integrated immunomodulators combined with immune checkpoint inhibitors is an ideal tool for stimulating effective immune response (Fig. 2c).

## Applications of nanotechnology to radioimmunology

**Fig. 3:**
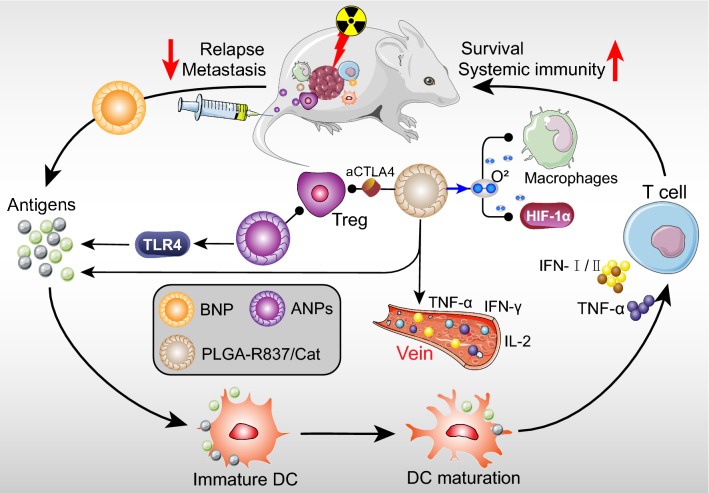
Representation of the nanoparticle and radiotherapy in systemic antitumor immunity. Three types of NPs (BNP, ANPs and PLGA-R837/Cat) combined with radiotherapy can promote the tumor associated antigen secretion, anti-tumor cytokines secretion and systemic immunity. The function of NPs not only stimulated the DCs and T cells activation, but also suppressed the tumor malignant immune cells such as Tregs and macrophages

Recently, RT combined with immunotherapies have attracted substantial attentions. Conventional RT is a classic type of local tumor treatment, however, distantly spreading and metastases cannot be controlled [159]. Therefore, developing next generation RT strategies is urgent for systemic clinical outcomes, such as combining with chemo-immunotherapy to improve tumor immune microenvironment [160, 161]. Generally, RT can induce tumor cells to release tumor-associated antigens, which triggered anti-tumor immune responses [162], yet systemic anti-tumor immune responses are rarely induced by RT alone [163]. Herein, the strategy of multifunctional NPs combined with RT to enhance immune responses was proposed, which not only control the local tumors, but also inhibit distant metastases and tumor relapse (Fig. 3) [163–165]. While, the combination of this strategy and immunotherapy to treat cancer is still under investigation [162, 166].

Patel et al. [163] designed a bacterial membrane-coated nanoparticle (BNP), consisting of an immune activating PC7A/CpG polyplex core coated with bacterial membrane and imide groups. This multi-component BNP had the ability to improve antigen retrieval, and enhanced-antigen finally interacted with MHC-I to boost innate immunity, such as more efficient and proliferation of T cells [167, 168]. Combined with RT, BNP can capture neoantigens and increase their absorption in DCs, then delivering to MHC-presented cells, finally facilitating the effector T-cell activation, tumor regression and antitumor immune memory compared to RT alone group [163, 169]. Acting as the adjuvant, PC7A and CpG modulated the antigen uptake in DCs. Compared to RT or BNP alone, BNP plus RT elevated MHC-I expression in TME and leaded to effector T cells and Type I IFN augment, which is necessary for effector T-cell recognition in tumor. Mice bearing NXS2 neuroblastoma with BNP plus RT also presented 100% complete regression of the primary tumors, leading to higher survival rate and lower metastasis rate [163]. Moreover, to overcome some negative feedback and ensure treatment effect, combining BNP plus RT with immune checkpoint blockades can inhibit immune suppressive signaling [170, 171]. Consequently, this breakthrough approach of RT combined with immunotherapy may systematically ameliorate tumor immunity and long-term immune response.

Furthermore, natural Herb Astragalus (APS) has been considered to enhance adaptive immune response, and APS polysaccharide NPs (ANPs) was synthesized with average size of 126 ± 1 nm, and zeta potential of − 8.09 ± 0.51 mV [164, 172]. Through enhancing Toll-like receptor 4 (TLR4) pathway related antigen presentation, ANPs regulated the maturation and activation of DCs, ultimately initiating T-cell expansion and antigen-specific immune response [164]. Within the treatment of ANP plus RT, ANP can reverse RT decreased population of CD8^+^ T-cell, increase TIL percentage, and decrease Tregs number. In ANPs plus RT group, CD4^+^ T/Tregs and CD8^+^ T/Tregs ratio were notably increased compared to control or RT alone, implying that can enhanced regional and systematic anti-tumor ability and improved tumor immune environment [164]. Impressively, survival rate of the ANP plus RT group was further prolonged, and the treatment group possessed a higher median survival than control (29.2 vs 24.8 days) [164]. Moreover, Li et al. indicated that diselenide-pemetrexed (Pem/Se) self-assemblies had diameters of 47 ± 2 nm. Under 2 Gy or 5 Gy γ-radiation, treatment with Pem/Se had higher level of ROS, resulting in the up-regulated apoptosis of cancer cells. Human leukocyte antigen E (HLA-E) was expressed on the membranes of several types of cancer cells. And diselenide bonds could be broke to form selenic acid under γ-radiation, which can inhibit HLA-E protein expression, ultimately up-regulating cancer immunity of NK cells. While, HLA-E protein expression was more inhibited after the combination treatment of Pem/Se and γ-radiation compared with Pem/Se treatment alone [173]. This combination therapy also up-regulated the level of IFN-γ and TNF-α, which confirmed the efficacy of cancer immunotherapy [173]. In addition, a nanomedicine (PSeR/DOX), composed of diselenide-containing polymer backbone, DOX and tumor-targeting peptide-modified polyethylene glycol (PEG-RGD), was designed to achieve chemotherapy, radiotherapy, and immunotherapy simultaneously. Through the EPR effect, PSeR/DOX NPs accumulated in tumor tissues. And the release of DOX from the NPs was induced by radiation, thereby enhancing the chemotherapy efficiency. In line with this, PSeR/DOX NPs combined with 5 Gy radiation remarkably increased the cytotoxicity and cell apoptosis ratios. Moreover, both PSeR/DOX NPs and PSeR/DOX NPs combined with 5 Gy radiation down-regulated HLA-E expression and boosted the the NK cell-mediated immunotherapy. However, the concentrations of IFN-γ was markedly increased in the combined treatment compared to other groups [174]. Collectively, multifunctional NP combined with RT was a novel strategy to enhance anti-tumor immunity.

Nowadays, the combination of RT-immunotherapy with multifunctional NPs also have broad development potential and synergistic therapeutic effects. To overcome tumor hypoxia-associated radiation resistance, Chen et al. [162] designed a multifunctional core–shell PLGA NP, which can simultaneously deliver Cat and hydrophobic imiquimod (R837: Immune adjuvant). PLGA-R837/Cat NPs composed a uniform sphere with a particle size of approximately 100 nm. As recent reported, after RT, PLGA NPs will capture tumor-associated antigens to enhance immune responses in relatively low cure rate (20%) [175], which may due to the hypoxia-associated radiation resistance [176]. Tumor hypoxia was suggested to increase the numbers of tumor-associated macrophages and promoted tumor cells progress [177]. Since Cat has the ability to decompose H_2_O_2_ into O_2_, after PLGA-R837/Cat injection of colon tumor, lower hypoxic probe (pimonidazole) and hypoxia-inducible factor (HIF)-1α signaling were detected [162]. Notably, with the radiation treatment, data indicated that PLGA-R837/Cat further promoted DCs maturation and tumor-associated antigens secretion than PLGA-R837 or free R837. However, PLGA or PLGA-Cat without R837 had no obvious effect on DCs. Therefore, PLGA-R837/Cat NPs combined with RT can synergistically enhance the tumors immune stimulation. In aspect of systemic immunity, the levels of mouse serum cytokines (IL-12, IFN-γ and TNF-α) in PLGA-R837/Cat plus RT group were highest among all test. These cytokines played a positive role in anti-tumor immunotherapy and abscopal anti-metastasis effect [178, 179]. Strikingly, this approach combined with CTLA-4 blocking (anti-CTLA4) can inhibit immune-suppressive Tregs in tumors and was favorable for anti-tumor immunity. PLGA-R837/Cat-based RT plus anti-CTLA4 induced effective long-term immune memory protection against cancer recurrence, sustaining high level of TNF-α and IFN-γ [162]. In addition, it not only induced effector memory T-cell, cytotoxic T lymphocytes and helper T-cell infiltration, but also effectively abrogated the activity of Tregs to promote anti-tumor immunity. Not surprising, through improving the anti-tumor immune responses, RT with PLGA-R837/Cat effectively inhibited tumor growth and recurrence, and immunomodulator loaded in this therapy can trigger stronger systemic immune responses to completely eliminate primary and/or distant tumors. As a result, it can extend the survival of 60% of the mice to 60 days, compared with 40 days of control groups. Overall, combined nanotechnology and radio-immunotherapy can promote the tumor associated antigen secretion, anti-tumor cytokines secretion and systemic immunity, which has the potential for clinical translation [162].

## Conclusions

Advancements in nanotechnology have been effectively developed in cancer therapy. High-Z metal NPs (including Au, Hf, Bi and Gd) and nano-delivered radiosensitizers (cisplatin and selenium) have become classically adjunctive treatments for cancers. The combination of nanomedicine and RT has greatly improved the efficacy of treatment for cancers and may obtain clinical translation. With the same momentum, recent advances in immune checkpoint blockade therapy have achieved remarkable results by the relevant nanotechnology, such as nano-encapsulated immune checkpoint inhibitor, nano-delivery PDL1 siRNA, and the combination of immune checkpoint inhibitor and nanoparticle-encapsulated different drugs. Simultaneously, the strategy of combining immunotherapy using nanotechnology with radiation therapy has proved effectively and has great potential for clinical translation. These findings will help instruct the deviser and exploitation of nanomedicine with ideal functions for clinical applications. Despite such enthusiasm, there are still challenges. It is unclear whether nanomaterials-activated immunity will over-activate immunity or boost the side effects of autoimmunity. In the future, it is expected to develop personalized novel theranostic NPs, which might obtain clinical translation and make great contributions to individual optimal treatment.

## Data Availability

All data generated or analyzed during this review are available from the corresponding author upon reasonable request.

## Abbreviations

NPs: Nanoparticles
EPR: Enhanced permeability and retention
RT: Radiotherapy
TME: Tumor microenvironment
IMRT: Intensity modulated RT
IGRT: Image guided RT
SABR: Stereotactic ablative RT
BSA: Bovine serum albumin
MSCs: Mesenchymal stem cell
Tregs: Regulatory T cells
mAbs: Monoclonal antibodies
Au: Gold
Hf: Hafnium
Bi: Bismuth
Gd: Gadolinium
AuNPs: Gold NPs
IR: Ionizing radiation
gAuNP: Goserelin-conjugated AuNP
TNF-α: Tumor necrosis factor-α
GPMs: Gold-loaded polymeric micelles
STS: Soft tissue sarcoma
EBRT: External beam RT
ROS: Reactive oxygen species
F-RBC: Folate plus red blood cell membrane
SiBiGdNP: Silica-based bismuth gadolinium NPs
MR: Magnetic resonance
ILS: Increased of life span
miRNA: MicroRNA
Se: Selenium
FDA: Food and Drug Administration
PSOC: Platinum-sensitive ovarian cancer
PROC: Platinum resistant ovarian cancer
H2O2: Hydrogen peroxide
SeNPs: Selenium NPs
PLGA-PEG: Polylactic acid-glycolic acid copolymer-polyethylene glycol
ACT: Adoptive cell therapy
PD-1: Programmed cell death protein 1
PD-L1: Programmed cell death-ligand 1
CTLA-4: Cytotoxic T lymphocyte antigen-4
IDO: Indoleamine 2,3-dioxygenase
CD40: Cluster of differentiation 40
CTLs: Cytotoxic T lymphocytes
aPD1: Anti-PD-1
Zeb: Zebularine
MDSCs: Myeloid-derived suppressor cells
DINP: Dual-immunotherapy NP
siRNA: Small interfering RNA
irAE: Immune-related adverse events
1MT: 1-methyl-DL-tryptophan
NK: Natural killer
DOX: Doxorubicin
ICD: Immunogenic cell death
MSN: Mesoporous silica NPs
DCs: Dendritic cells
DMONs: Dendritic mesoporous organosilica NP
LCP: Lipid/calcium/phosphate
MMP-2: Matrix metalloproteinase-2
TEM: Transmission electron microscope
MHC: Major histocompatibility complex
BNP: Bacterial membrane-coated nanoparticle
APS: Natural Herb Astragalus
TLR4: Toll-like receptor 4
HIF: Hypoxia-inducible factor
MRI: MR imaging
OS: Overall survival
HLA-E: Human leukocyte antigen E
